# Ubiquitin and ubiquitin-like molecules in DNA double strand break repair

**DOI:** 10.1186/s13578-020-0380-1

**Published:** 2020-02-11

**Authors:** Jia Yu, Bo Qin, Zhenkun Lou

**Affiliations:** 1 Division of Clinical Pharmacology, Department of Molecular Pharmacology and Experimental Therapeutics, Mayo Clinic, Rochester, MN 55905 USA; 2 Department of Oncology, Mayo Clinic, Rochester, MN 55905 USA; 3 Gastrointestinal Research Unit, Mayo Clinic, Rochester, MN 55905 USA

**Keywords:** Double strand break, Ubiquitin, Ubiquitin-like protein, DNA damage, DNA repair

## Abstract

Both environmental and endogenous factors induce various forms of DNA damage. DNA double strand break (DSB) is the most deleterious DNA lesion. The swift initiation of a complexed network of interconnected pathways to repair the DNA lesion is essential for cell survival. In the past years, the roles of ubiquitin and ubiquitin-like proteins in DNA damage response and DNA repair has been explored. These findings help us better understand the complicated mechanism of DSB signaling pathways.

The human genome suffers either exogenous or endogenous damages all the time. Immediate and proper DNA damage response is essential for guarding genomic stability. Upon DNA damage, apical sensors are activated and transduce the signals to the downstream effectors through transducers. Ubiquitin and ubiquitin like proteins mediated posttranslational modifications play an important role in cellular response to these stresses to maintain genomic integrity.

## The response and repair of the DNA double-strand breaks

Once DSB happens, the master kinase ATM is activated and initiates global signaling cascades and enriches the DNA damage signaling and repair factors at the DSB sites [1–6]. ATM kinase is recruited to DSB through interaction with the C-terminus of NBS1, which is a subunit of the DSB sensor- the MRN complex, in response to DSB [7–9]. It has been reported that MRN complex can stimulate ATM kinase activity directly in vitro [10, 11]. However, the exact mechanism by which MRN activates ATM is still not well understood. Activated ATM rapidly phosphorylates H2AX at serine 139, a variant of histone H2A, spreading in a bidirectional manner and spanning 1–2 Mb in mammals in the DSBs [12]. MDC1, the major reader for γH2AX, contains tandem BRCT domains at its C-terminus that specifically bind γH2AX at DSB sites [13, 14]. Oligomerization of MDC1 is critical for recruitment of MDC1 complex at DSBs [15–17]. Recruited MDC1 is phosphorylated by ATM and this phosphorylation is recognized by the FHA domain of the ubiquitin ligase RNF8 and recruits RNF8 to break sites [14, 18–21]. Meanwhile, MDC1 recruits more MRN complexes and ATM proteins, phosphorylating more H2AX at S139, spreading the assembly along chromatin and amplifying DDR signaling [14, 16, 17, 22–25]. RNF8, once recruited to the DSB, ubiquitinates the L3MBTL2 whose ubiquitination recruits another ubiquitin ligase, RNF168 to the damage site. RNA168 then ubiquitinates H2A at lysine 13/15 to promote engagement of 53BP1 and BRCA1 to DSBs [26–29]. DSBs are mainly repaired through two mutually exclusive pathways: homologous recombination (HR) and nonhomologous end joining (NHEJ) [23, 30]. Once a break is detected, 53BP1 and BRCA1 compete for directing the cell to commit to NHEJ or HR respectively [31]. CtIP, which is stimulated by BLM, MRN and RPA with enhanced endonuclease activity, cooperates with the nucleases EXO1 and BLM/DNA2 to resect the DNA [32–36]. This process creates extensive single-stranded (ss) 3′ DNA overhangs. Replication protein A (RPA) wraps these ssDNAs, thereby protecting it from breakage [32, 37]. Subsequently, BRCA1-PALB2-BRCA2 complex with DSS1 promotes the replacement of RPA and the loading of the recombinase RAD51 onto ssDNA to form the pre-synaptic filament [38, 39]. RAD51 nucleoprotein filaments search for a homologous sequence to invade and displace one strand of the homologous template to form a displacement loop, facilitating sister chromatid exchange [40–43]. To inhibit end resection induced homologous recombination, 53BP1 interacts with RIF1, the Shielding complex (REV7–SHLD1–SHLD2–SHLD3) and the CST/Pol α-Prim complex, and recruitment of these complexes shields DNA end from resection, and promote NHEJ [23, 44–48]. UHRF1, an E3 ligase, is also involved in this process. We discovered that UHRF1 mediates K63-linked polyubiquitination of RIF1, and leads to its dissociation from 53BP1 and DSBs, thereby regulating DSB repair choice [49]. In this review, we have summarized the role of ubiquitin and ubiquitin like modifiers (Table 1) in response to DNA double strand breaks (DSBs) in mammalian cells.

**Table 1:** Summary of the ubiquitination and ubiquitination like systems

Modification	Modifier encoding gene	Mature modifier protein	E1	E2	E3	Active protease
Ubiquitination	UBB, UBC, UBA52 and RPS27A	76 amino acids	2 (UBA1 and UBA6)	~ 40	Over 600	Dozens (USPs,UCHs,OTUs,MJD,JAMM)
Sumoylation	SUMO1-4	varies from SUMO1-4	1 (SAE)	1 (UBC9)	A dozen	6 (SENPs)
Neddylation	NEDD8	76 amino acids	1 (NAE)	2 (UBC12 and UBE2F)	Over 600	3 (UCHL1, UCHL3 and USP21)
Ufmylation	UFM1	83 amino acids	1 (UBA5)	1 (UFC1)	1 (UFL1)	1 (UFSP2)
Isgylation	ISG15	163 amino acids	1 (UBE1L)	1 (UBCH8)	2 (HERC5 and TRIM25)	5 (USP18, USP2, USP5, USP13 and USP14)
Fatylation	FAT10	165 amino acids	1 (UBA6)	1 (USE1)	Unknown	Unknown

## Ubiquitin dependent signaling response to DSB

Ubiquitin (Ub) is a highly conserved small protein, composed of 76 amino acids. Ubiquitination is the process of addition of ubiquitin protein to its substrate through a cascade of reactions. This process includes three steps reaction. First, E1 the Ub-activating enzyme activates Ub with adenosine triphosphate and transfers it to E2 Ub-conjugating enzyme. Second, C-terminal carboxyl group of Ub forms a thioester bond with active cysteine in E2 protein. Third, the E3 Ub ligases (E3s) catalyze the C-terminal carboxyl group of Ub, forming of an iso-peptide bond with the lysine ε-amino group of the substrate [50, 51]. More than 600 E3 ligases are predicted in human genome and these E3 ligase are categorized into two classes: the RING domain or U-box containing E3s (promoting direct Ub transfer from an E2 to a substrate), and the HECT (homologous to the E6AP carboxyl terminus) containing E3s (directly catalyze the covalent attachment of ubiquitin to substrate proteins) [52]. The substrate can be modified with one ubiquitin or multi-ubiquitin formed polyubiquitin chain at one or several lysine residues in the substrate protein. In the ubiquitin polypeptide, there are seven lysines: K6, K11, K27, K29, K33, K48 and K63. Ubiquitin moieties can be conjugated through one of the lysine residues or the N-terminal methionine residue to form specific polyubiquitin chain [52]. Different ubiquitin linkage could induce different functional outcomes. Usually, the substrate modification by Lys-48- or Lys-11-linked polyubiquitin chain is powerful degradation signal, which is recognized by proteasome, thus leading to substrate degradation. Whereas, the Lys-K63 or other different lysine linked polyubiquitin chain modification induces the change of the activity or cellular localization of its substrate [53]. In addition, single ubiquitin modified substrates are also frequently reported.

Ubiquitination of histone proteins is an important step in DNA damage response. The nucleosomes are composed of four core histones, H2A, H2B, H3 and H4, and linker histone H1. Following double strand break, H2A is ubiquitinated on K13 and K15 by RNF168. Both K127 and K129 can be ubiquitinated by BRCA1/BARD1 [27, 54, 55]. L3MBTL2 is tethered by MDC1 to the vicinity of the DNA lesion and K63-linked polyubiquitinated by RNF8. Ubiquitinated L3MBTL2 is subsequently recognized by RNF168 and recruited to DSBs. RNF168 then ubiquitinates proteins such as histone H2A and H2AX to further amplify the damage response and recruit repair proteins such as BRCA1 and 53BP1 [29]. The E3 ligase RNF168 primes mono-ubiquitination of H2A at lysine 15, and RNF8 extends the monoubiquitination on this site to help RNF168-mediated K63-linked polyubiquitination at lysine K15 subsequently once double strand breaks happen. RNF168 binds modified H2A by itself and elevates its concentration at the DSB and further amplifies H2A monoubiquitination signal [27, 56, 57]. This monoubiquitination signal and dimethylation of H4K20 is recognized by 53BP1 and recruitment of 53BP1 inhibits DNA end resection, thus suppressing HR and promotes NHEJ [58–60].

Protein ubiquitination is reversible and dynamic and the level of protein ubiquitination is balanced by E3 ligase and deubiquitinase. Deubiquitinase is responsible for removing ubiquitin from the substrate and regulating protein stability, activity or cellular localization. Till now, five DUB families have been discovered, including ubiquitin-specific proteases (USPs), ubiquitin C-terminal hydrolases (UCHs), ovarian tumor proteases (OTUs), Machado–Joseph disease (MJD) protein domain proteases, and JAMM motif zinc metalloproteases [61].

The ubiquitination of H2A at lysine 13/15 is deubiquitinated by USP51 and USP3 and loss of USP51 causes increased 53BP1 foci formation, and boosted sensitivity to IR [62, 63]. Other deubiquitinases are also suggested to remove ubiquitin at both sites, for example, USP44, and USP11 [64, 65]. JAMM deubiquitinase family member BRCC36 complex with BRE or NBA1 opposes RNF8 mediated ubiquitination of H2AX and enhances radiosensitivity [66–70]. The ubiquitination of H2A at lysine 127/129 is performed by BRCA1/BARD1 E3 ligase complex and recruits SMARCAD1 for 53BP1 reposition, enhancing end resection and HR [55, 71]. PALB2 is also important for HR through interaction with BRCA1, forming complex with RAD51 and BRCA2 and facilitates RAD51 replacement of RPA [72–75]. The ubiquitination of BRCA1 interacting partners PALB2, which is mediated by KEAP1 E3 ligase complex, decreases its interaction with BRCA1 and suppresses HR in G1 phase [76]. The deubiquitinase, USP11, counteracts this process by removing polyubiquitin chain from PALB2 and sabotages the BRCA1-PALB2-BRCA2 complex assembly [76]. RNF169 is recruited to DSBs through its ubiquitin-binding MIU2 domain, competes with RNF168, removes 53BP1 and RAP80 bridging ubiquitin and histone surfaces, and promotes HR [77–79]. Ubiquitination of RAD51 also impairs RAD51-BRCA2 interaction, and UCHL3 mediated deubiquitination of RAD51 strengthens RAD51-BRCA2 binding and facilitates RAD51 recruitment to DSBs [80].

Another core histone H2B is globally monoubiquitinated at lysine 120 in response to DNA damage mediated by RNF20-RNF40 E3 ubiquitin ligase complex. Monoubiquitinated H2B leads to chromatin relaxation, thereby increasing the accessibility of DNA repair factors following DNA damage [81]. Interestingly, acetylation of H2BK120 is found at 1 KB window of DSB and monoubiquitination of H2BK120 is beyond that window [82]. The function of H2B ubiquitination is still not clear. It might be important for DNA damage checkpoint response and HR [83, 84]. USP22 and the SAGA complex are reported to remove the ubiquitin from H2B and regulate early stage of DNA damage response.

The recruitment of both 53BP1 and BRCA1 to DSB sites is through RNF8/RNF168 mediated ubiquitination, despite of their different functions in DNA repair. 53BP1 recognizes both H4K20me2 and H2A K15ub at DNA damage sites, whereas the recruitment of BRCA1 is depending on RAP80 mediated by its ubiquitin-binding modules (UIMs) binding K63-linked ubiquitin chains on chromatin [18, 19, 85]. The ubiquitination of RAP80 status affects its cellular localization in the cells. USP13, translocated to the DSBs after IR, deubiquitinates RAP80, enhances the recruitment of RPA80-BRCA1 complex to break sites [86]. BRCA1/BARD1 serves as an E3 for a range of substrates at DNA damage sites, including CtIP [87]. CtIP ubiquitination by BRCA1 does not mediate the degradation of CtIP protein. Actually, ubiquitinated CtIP facilitates its chromatin loading and plays an important role in regulating of G2/M checkpoint [87]. Recently, two groups reported that USP4 deubiquitinates CtIP, facilitates CtIP to the damage sites, and enhances HR [88, 89].Further studies are needed to explore the complicated relationship between both two proteins as well as the determinants for DSB repair pathway choices.

## Sumoylation dependent signaling response to DSB

Small ubiquitin-like modifiers (SUMOs) mediated modification is essential for eukaryotes [90]. Four SUMO genes are discovered in vertebrate genomes, including SUMO1-4 [91]. SUMO2 and SUMO3 polypeptides are very similar, and they share 97% sequence similarity in human. However, SUMO1 is quite different (47% similarity shared by SUMO1 and SUMO2) [90]. Recently, SUMO4 is identified, sharing high similarity with SUMO2 and SUMO3. However, it contains a unique proline residue located at position 90, preventing its maturation by SUMO protease [92, 93]. With high similarity to ubiquitin, SUMO is also a small protein and can be conjugated to the target proteins through a serial enzymes, including E1 (SUMO-activating enzyme), E2 (SUMO-conjugating enzyme), and E3 (SUMO ligase) [94]. However, the number of these enzymes is much less than ubiquitin system. Till now, only 1 E1 and 1 E2 and around a dozen of known E3s. Like ubiquitination, sumoylation can be reversed by sentrin-specific proteases (SENPs) including SENP1, SENP2, SENP3, SENP5, SENP6 and SENP7 [91].

SUMO plays important roles in the DNA damage response. Following DNA damage, numerous of DNA repair factors are sumoylated. SUMO1-3 aggregate at DSBs in human cells and SUMO ligase PIAS1 and PIAS4 are required for this accumulation of these SUMO proteins, which are important for subsequent robust ubiquitination of DSB-flanking chromatin mediated by RNF8, RNF168, and BRCA1 [95]. Later on, another study further found that sumoylation of HERC2 by PIAS4 increases its interaction with RNF8 in a DNA damage dependent manner and stabilizes the RNF8-Ubc13 complex [96]. These studies also suggest a more upstream role of SUMO in this response. RAP80 binding to SUMO at DNA damage sites via a SUMO-interacting motif (SIM) is also important for its recruitment to DSB sites [97, 98].

BRCA1 is also found to be sumoylated by PIAS SUMO E3 ligase in response to DSB, enhancing the ubiquitin ligase activity of BRCA1/BARD1complex and promoting HR [99]. CBX4 sumoylates BMI at lysine 88 and facilities the recruitment of BMI to the damage sites, mediating different DNA damage response pathways from PIAS1/PIAS4 [100]. Sumoylation is also critical for turning off DNA damage response after DNA repair is completed. Several labs independently reported that MDC1 is sumoylated at the damage site. Sumoylated MDC1 is recognized by the SUMO interacting motifs (SIM) of RNF4 and is further ubiquitinated by RNF4, inducing its degradation and removal of MDC1 and 53BP1 from DSBs [101–103]. In addition, RNF4 also interacts with other SUMOylated protein through its SIMs and ubiquitinates polysumoylated proteins in order to facilitate their degradation [103].

SUMO and ubiquitin can form hybrid chains. The hybrid chains are recognized by proteins which contain tandem SUMO- and ubiquitin-interacting motifs (tSIM-UIMs), such as RAP80. The tSIM-UIMs in RAP80 enable it to interact with greater affinity to hybrid SUMO-Ub chains compared with homotypic SUMO or ubiquitin chains. At damage sites, RNF4, a SUMO-targeted ubiquitin E3 ligase, catalyzes SUMO-Ub hybrid chains. These hybrid chains are recognized by RAP80, thus promoting BRCA1 accumulation and enhancing HR [97, 98]. The protease for removing the hybrid chains is still not clear.

## Neddylation dependent signaling response to DSB

Neural Precursor Cell Expressed Developmentally Down-Regulated Protein 8 (NEDD8) is another ubiquitin like protein with 81 amino acids (NEDD8 precursor). DEN1 processes NEDD8 to its mature form by removing the last 5 amino acids from its C-terminal tail [104]. One family of the E3 ligases- Cullin-RING ligases (CRLs) requires covalent modification of its core cullin protein with NEDD8 to enhance their ligase activity [105]. NEDD8 is conjugated to its target proteins by an enzymatic neddylation cascade, including E1-activating enzyme (NAE) and E2-conjugating enzymes (UBC12, UBE2F) [106, 107]. NAE binds MgATP and NEDD8 and forms acyl adenylated NEDD8. Acyl adenylated NEDD8 subsequently reacts with the active thiol site of the enzyme to form a NEDD8-NAE thioester, coupled with the release of AMP. Another reaction generates a second acyl adenylated NEDD8, forming a ternary complex capable of transferring NEDD8 to E2. The last step is transferring NEDD8 from E2 to the respective cullins [108].

Neddylation functions critically in DNA damage response. CUL1 substrate receptor SKP2 modifies NBS1 with lysine 63 polyubiquitin chain and enhances its interaction with ATM, thus promoting ATM activation upon DNA damage happens [109]. RNF111/UBE2M-mediated neddylation inhibits BRCA1 and CtIP-mediated DNA end resection, and regulates the choice between NHEJ and HR and also the balances between different recombination sub-pathways [110]. Interestingly, RNF168 is reported to function as a NEDD8 E3 ligase after DNA damage [111]. It mediates both H2A ubiquitination and neddylation and the two modifications are against each other. RNF168 itself is also neddylated and this neddylation enhances its interaction with its E2 UBC13 [111]. CDC25A is a member of the CDC25 family of phosphatases and required for activation of cyclin-dependent kinases and cell cycle progression from G1 to the S phase. CDC25A is phosphorylated by CHEK2 following IR and ubiquitinated by CRL E3 ligase for degradation, leading to the cell cycle arrest, which leaves cells sufficient time to repair DNA damage [112]. Removal of NEDD8 from its substrate is also important. COP9 signalosome, a protein complex comprising 8 subunits, is in charge of deneddylation of CRLs [113]. NEDD8 is erased by the metalloprotease activity of a subunit COP9 signalosome-COPS5, thereby suppressing the CRLs [114]. COPS5 is found to be important for Rad51 protein stability. Suppression of COPS5 induces degradation of Rad51 protein and inhibition of HR due to enhanced CRL E3 ligase activity [115].

## Ufmylation dependent signaling response to DSB

Ubiquitin fold modifier 1 (UFM1) is also a member of ubiquitin like proteins [116]. Similar to other modifiers, UFM1 protein needs further maturation by removing the last two amino acids in mammalian cells and exposing glycine residue for subsequent conjugating reactions. Parallel to ubiquitination system, ufmylation system also includes E1, the UFM1-activating enzyme (ubiquitin-like modifier-activating enzyme 5; UBA5), E2, the UFM1-conjugating enzyme 1 (UFC1); and E3, the UFM1-specific ligase 1 (UFL1). In contrast to numerous E3s for ubiquitination, only one E3 (UFL1) is identified so far. UFM1 can be conjugated to its substrates via these three enzymes: UFM1 is activated by UBA5 in the presence of ATP and undergoes thioester reaction with the Cysteine 250 of UBA5. Conjugated UBA5 interacts with UFC1 and transfer UFM1 to UFC1, forming a similar thioester linkage with Cysteine 116 in UFC1. The E3 ligase UFL1 transfers UFM1 from UFC1 to its target proteins [116]. The ufmylation is also a reversible process. UFM1 can be cut off from its substrate by the specific UFM1-specific proteases (UFSP). Again, in human, only one functional UFSP protein called UFSP2 has been identified so far [117]. Previous studies have suggested ufmylation system play important roles in hematopoiesis, reticulum homeostasis, liver development, mitosis, gene transcription, G-protein coupled receptor (GPCR) biogenesis, and fatty acid metabolism [118–124]. Genome-wide association studies also suggest UFM1 signaling is associated with a number of human diseases, including cancer, ischemic heart diseases, diabetes, atherosclerosis, hip dysplasia, and schizophrenia [125–128]. Recently, our group and others have discovered its new role in ATM activation following DNA damage [129, 130].

MRN complex serves as an initiator of DNA damage response and DNA repair. It is responsible for ATM recruitment to DSBs. One study suggest that MRE11 is ufmylated, and this modification is important for the stability of MRN complex and ATM activation [130]. Our group also found that ufmylation signaling is important for ATM activation. We found that UFL1 aggregated at break sites quickly after double-strand breaks in an MRN complex dependent manner [129]. Recruited UFL1 mono-ufmylates histone H4 at Lysine 31, which helps recruiting Suv39H1-KAP1-HP1 complex to the DSBs, resulting in H3K9 trimethylation and the activation of Tip60-ATM pathway [129]. Interestingly, activated ATM phosphorylates UFL1 at serine 462 and elevates UFL1 activity, forming a positive feedback loop and amplifying ATM activation signal. These studies suggest a tight interplay between the DDR and the UFM1 pathway [129].

## Isgylation dependent signaling response to DSB

Interferon-stimulated gene 15 (ISG15) is a 17 kd protein and becomes mature until 8 amino acids are removed from its C terminus [131]. Type I interferon and virus infection stimulate its expression [132]. Like ubiquitination and other ubiquitin like modification, Isgylation also requires cascades of enzymes: an E1 (ubiquitin-activating enzyme E1-like protein, Ube1L; also named as UBA7), an E2 (ubiquitin-carrier protein H8, UbcH8), and an E3 [HECT domain and RCC1-like domain-containing protein 5 (HERC5), and TRIM25 in humans] [133]. ISG15 is activated by E1 enzyme UBE1L, forming a high-energy thioester intermediate. Then it is transferred to the active-site cysteine of E2 UbcH8. E3 ligases HERC5 subsequently transfers the activated ISG15 to the substrates [134, 135]. USP18, USP2, USP5, USP13 and USP14 are the ISG15-specific proteases and unconjugate ISG15 from its substrates [136–139]. DNA damage inducers induce Isgylation of p53 at K291 and K292 by the E3 ligase EFP and greatly enhances p53 transcriptional activity; hence the transcription of p53 target genes (CDKN1, BAX, MDM2 and ISG15) and Isgylation factors are elevated. Isgylated p53 also facilitates its phosphorylation and acetylation, thus suppression of cell growth and tumorigenesis [140]. Another DNA damage response protein, PCNA also can be modified by ISG15 [141].

## Fatylation dependent signaling response to DSB

Ubiquitin like protein HLA-F adjacent transcript 10 (FAT10) is encoded in the major histocompatibility complex class I locus and is synergistically inducible with interferon-γ and tumor necrosis factor α. FAT10 is different from other members. Its protein contains two ubiquitin like domain [142] and a free GG motif located at its C terminal tail [143]. Due to its unique structure, it is immediately available for activation and conjugation. In FAT10 conjugation cascade, FAT10 binds to its E1 UBA6 and forms UBA6-FAT10 thioester. E2 protein USE1, also named UBE2Z, transfers the thioestered FAT10 to its lysine to form a stable isopeptide linkage [144]. The E3 ligase for FAT10 and the deconjugating enzymes have not been discovered yet.

The function of FAT10 has previously been suggested. Depletion of FAT10 in mice prolongs lifespan and reduces adiposity, thus suggesting that FAT10 has a role in aging [145]. Its aberrant expression have been investigated in various cancer types, such as gastrointestinal cancer, hepatocellular carcinoma (HCC), pancreatic ductal adenocarcinoma, human glioma and cervical cancer [146]. The role of FAT10 in DNA damage response is not very clear. Only limited studies have been performed. Proteomic analyses of Fatylation have identified many DNA damage response proteins as FAT10 substrates, including Ku70, RECQ1, FUS, RAD51C, PCNA, DNAJA1, H2AX, KAP-1 [147, 148]. Recently, it is confirmed that IR induces the increase of Fatylated PCNA and leads to degradation of PCNA [149]. More studies are required to better understand the role of FAT10 in DNA damage response.

## Conclusions

Impressive studies of the principle and mechanism of ubiquitination, and ubiquitination- like modifications in DSB induced DNA damage response and DNA repair have been published. Here we listed the targets of ubiquitination, sumoylation, neddylation, Isgylation, and ufmylation, which are involved in the DSB signaling. We expect that the list of the substrates of those modifications will keep growing. Many challenges are still present. Even though ubiquitination and sumoylation are extensively studied, new question is emerging. For example, what is the function of branched Ub chain and Ub chain with hybrid linkage? Recent studies also suggest that Ub itself can be modified by PTMs, such as phosphorylation, and how these PTMs affecting Ub reaction and substrate function is not entirely clear. In addition, the exact function of ISG15 and FAT10 is still not clear. For some other Ub like molecules, such as ubiquitin-related modifier-1 (URM1), fan ubiquitin-like protein 1 (FUB1) and histone mono-ubiquitination 1 (HUB1), their functions in DNA damage have not yet been explored. Further studies are therefore needed to explore new principles and mechanism of these new ubiquitin like proteins in maintenance of genome stability. Further efforts are also likely to study the crosstalk among these modifiers and their contribution to DSB induced DNA damage response. A deep understanding of the principle and mechanism of these posttranslational modifications can also potentially provide new therapeutics for the cancer patients. The inhibitor of Neddylation E1 enzyme NAE—MLN4924 has been developed and displays dramatic inhibition of hematological malignancies and solid tumors [150–161]. Multiple Phase I clinical study of MLN4924 are undergoing [162–166]. Better understanding of the complexity of these signaling will help develop more small molecular inhibitors to target the factors in uibiquitination and ubiquitination like modification pathways for treating cancer or other diseases.

## Abbreviations

DSB: DNA double strand break
HR: Homologous recombination
NHEJ: Nonhomologous end joining
Ub: Ubiquitin
SUMO: Small ubiquitin-like modifier
NEDD8: Neural precursor cell expressed developmentally down-regulated protein 8
UFM1: Ubiquitin fold modifier 1
ISG15: Interferon-stimulated gene 15
FAT10: HLA-F adjacent transcript 10
URM1: Ubiquitin-related modifier-1
FUB1: Fan ubiquitin-like protein 1
HUB1: Histone mono-ubiquitination 1
